# Protective Effect of Vitamin K2 (MK-7) on Acute Lung Injury Induced by Lipopolysaccharide in Mice

**DOI:** 10.3390/cimb46030110

**Published:** 2024-02-22

**Authors:** Weidong Yang, Yulian Wang, Lulu Liu, Lihong Liu, Shuzhuang Li, Yuyuan Li

**Affiliations:** 1 College of Basic Medical Science, Dalian Medical University, Dalian 116041, China; czdywd@163.com (W.Y.); wangyulian240126@163.com (Y.W.); shdxlnyxyjy@163.com (L.L.); liulihong0416@163.com (L.L.); 2 Advanced Institute for Medical Sciences, Dalian Medical University, Dalian 116041, China

**Keywords:** vitamin K2, acute lung injury, apoptosis, tight junction injury, mitochondrial damage, autophagy

## Abstract

Vitamin K2 (MK-7) has been shown to cause significant changes in different physiological processes and diseases, but its role in acute lung injury (ALI) is unclear. Therefore, in this study, we aimed to evaluate the protective effects of VK2 against LPS-induced ALI in mice. The male C57BL/6J mice were randomly divided into six groups (n = 7): the control group, LPS group, negative control group (LPS + Oil), positive control group (LPS + DEX), LPS + VK2 (L) group (VK2, 1.5 mg/kg), and LPS + VK2 (H) group (VK2, 15 mg/kg). Hematoxylin–eosin (HE) staining of lung tissue was performed. Antioxidant superoxide dismutase (SOD) and total antioxidant capacity (T-AOC) activities, and the Ca^2+^ level in the lung tissue were measured. The effects of VK2 on inflammation, apoptosis, tight junction (TJ) injury, mitochondrial dysfunction, and autophagy were quantitatively assessed using Western blot analysis. Compared with the LPS group, VK2 improved histopathological changes; alleviated inflammation, apoptosis, and TJ injury; increased antioxidant enzyme activity; reduced Ca^2+^ overload; regulated mitochondrial function; and inhibited lung autophagy. These results indicate that VK2 could improve tight junction protein loss, inflammation, and cell apoptosis in LPS-induced ALI by inhibiting the mitochondrial dysfunction and excessive autophagy, indicating that VK2 plays a beneficial role in ALI and might be a potential therapeutic strategy.

## 1. Introduction

According to data from the World Health Organization, over 760 million people have been affected by and more than 6.9 million people have died due to SARS-CoV-2. The current trends indicate that “Long COVID” is potentially the next public health disaster that is brewing [1]. Unfortunately, there is still no effective treatment available against COVID-19. Systemic anti-inflammatory therapy is the most potent treatment. Corticosteroids, specifically dexamethasone (DEX), have been shown to decrease mortality in patients with severe COVID-19 who are hospitalized and require supplemental oxygen [2]. However, corticosteroids are not without their adverse effects, which include hyperglycemia, secondary infection, and psychiatric effects [3]. The high mortality rate due to this novel β-coronavirus SARS-CoV-2 infection resulted mainly from the development of acute lung injury (ALI) and its severe form, acute respiratory distress syndrome (ARDS) [4]. Therefore, it is necessary to develop an effective treatment strategy against ALI/ARDS to alleviate the serious threat of COVID-19 to human health.

Lipopolysaccharide (LPS), an extract of Gram-negative bacteria, can trigger severe lung injury, similar to COVID-19 [5]. Inhibiting the inflammation, apoptosis, and disruption of the lung epithelial and endothelial barriers is necessary for attenuating LPS-induced ALI [6]. Tight junction proteins (TJPs), such as zonula occludens-1 (ZO-1) and occludin, are important for maintaining TJ function [7]. Changes in the content of these proteins are considered to be of great significance in the physiological functions of the lung epithelium and endothelial barrier [8]. During ALI, pro-inflammatory cytokines could induce and aggravate barrier dysfunction by disrupting the expression of TJPs and increasing apoptosis [9]. Pulmonary barrier dysfunction further exacerbates inflammatory cell infiltration, suppresses inflammation, and exacerbates ALI progression. Therefore, inhibiting inflammation and cell apoptosis, and repairing the expression of intercellular TJPs are effective strategies for ALI treatment.

There is overwhelming evidence that mitochondrial dysfunction plays a vital role in the etiology of ALI [10,11]. Mitochondria are the site of oxidative metabolism in eukaryotes, which serve as scaffolds to initiate protein–protein signaling, and regulate Ca^2+^ homeostasis and reactive oxygen species (ROS) [12]. Mitochondria are also involved in autophagy, mitophagy, cell death signaling, and innate immune responses [13]. Oxidative damage to mitochondria stimulates the expression of PTEN-induced putative kinase 1 (PINK1), which mediates mitophagy, the removal of damaged mitochondria [14]. PINK1 phosphorylates mitofusin 2 (Mfn2), the main mitochondrial fusion protein, which recruits Parkin to the outer membrane [15]. The transcriptional activator PPARG coactivator 1 alpha (PGC-1α) is the main regulatory factor of mitochondrial biogenesis and therefore plays an important role in modulating mitochondrial quality control [16]. The mitochondrial health and functions rely on the accurate quality control of these proteins.

Autophagy is a process of self-degradation that enables the metabolic needs of cells themselves and the renewal of certain organelles [17]. And, deficiencies in these processes may lead to diseases, symptoms of which are significantly improved by regulating autophagy [18]. Autophagy is also involved in the development of ALI; however, its role in ALI is controversial, as it has been shown in different reports to positively or negatively regulate ALI [19,20].

To date, studies have shown that some vitamins (A, B6, B12, folate, C, D, and E) play a preventive role against SARS-CoV-2 infection [21,22]. Vitamin K (VK) is another natural fat-soluble vitamin comprising three types: VK1 (phylloquinone), VK2 (menaquinones or MK-n), and VK3 (menadione) [23]. VK2 generates from the activity of intestinal bacteria or the conversion of dietary VK1 [24]. VK2 is absorbed more readily than VK1, and among different MKs, MK-7 has the highest bioavailability [25]. In recent years, VK2 has become an important biologically active compound, due to its variety of biological functions, including anti-inflammatory [26], antioxidant stress [27], anti-apoptosis [28], and anti-autophagy [29] activities, in addition to repairing mitochondrial damage [30]. Therefore, based on the pathogenesis of ALI and the pharmacological effects of VK2 (MK-7), we hypothesized that VK2 has favorable effects on ALI.

## 2. Materials and Methods

### 2.1. Reagents

VK2 (MK-7) was provided by Sungen Bioscience Co., Ltd. (Guangzhou, China). LPS (from *Escherichia coli* (055:B5)) and DEX were procured from Sigma-Aldrich (St. Louis, MO, USA). The superoxide dismutase (SOD, BC0175) and total antioxidant capacity (T-AOC, BC1315) determination kits were purchased from Solarbio (Beijing, China). The calcium (Ca^2+^) assay kit (C004-2-1) was purchased from Jiancheng Bioengineering Institute of Nanjing (Nanjing, China). Primary antibodies were purchased from Abcam (Cambridge, MA, USA), Wanleibio (Shenyang, China), Proteintech (Wuhan, China), and HuaAn (Wenzhou, China). Other chemicals were reagent grade.

### 2.2. Animal and Experimental Protocol

The animal research was carried out in Dalian Medical University following protocols authorized by the Dalian Medical University Animal Care and Use Committee (AEE20051). Forty-two male C57BL/6J mice (8–10 weeks old, 20–24 g body weight) were purchased from Liaoning Changsheng Biotechnology Co., Ltd. (Shenyang, China). All mice were housed, five per cage, with a 12 h light/dark cycle and in a pathogen-free environment at 24 ± 2 °C and 55 ± 5% humidity, and they had free access to food and water. The mice were allowed to acclimatize for 1 week prior to the experiments. Six groups of mice were split at random (n = 7 each): control group, LPS group, negative control group (LPS + Oil), positive control group (LPS + DEX), and the VK2 treatment groups at two different concentrations.

**Figure 1:**
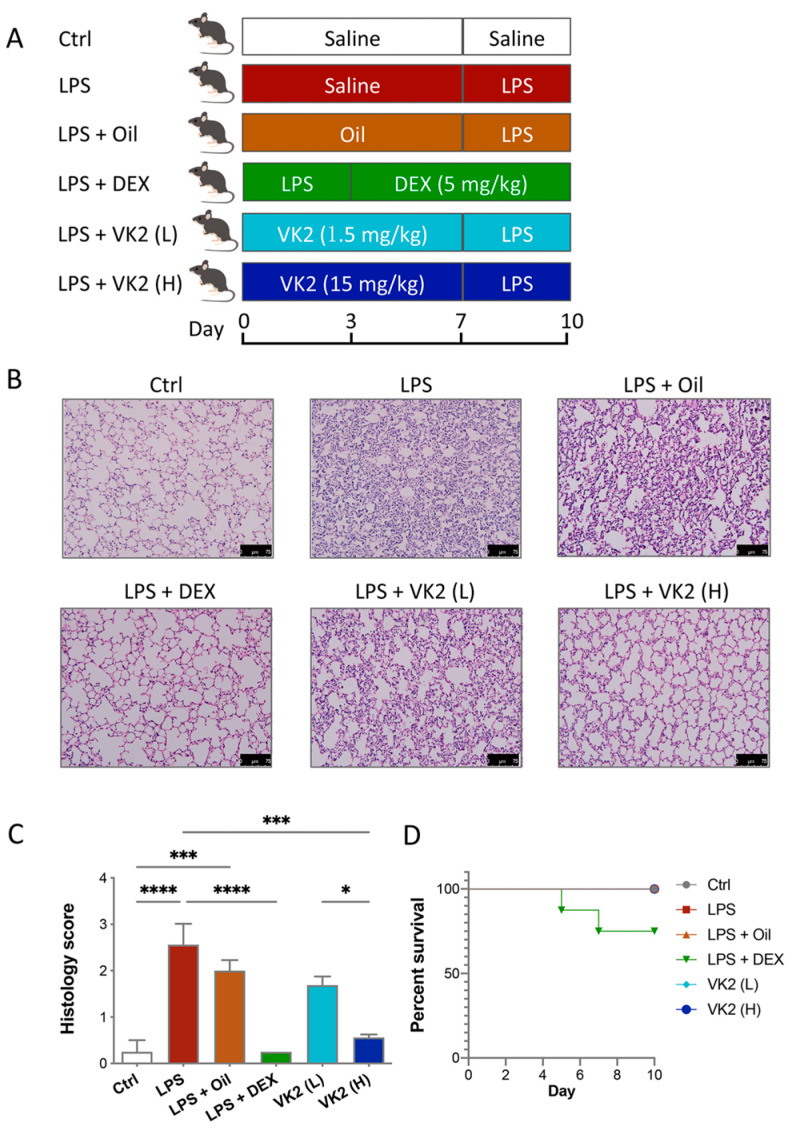
VK2 alleviated LPS-induced acute lung injury in mice. (**A**) Mice were pre-administered a solvent or vitamin K2 (1.5 and 15 mg/kg, respectively), followed by intraperitoneal injection of lipopolysaccharide (7 mg/kg). (**B**) Hematoxylin–eosin staining (original multiple: 200×, scale = 75 μm) was performed on paraffin sections of lung tissue for histological scoring. (**C**) Lung tissue injury in each group was evaluated by histological score. (**D**) The survival curves of ALI mice treated with DEX and VK2. Values represent means ± SEM; * *p* < 0.05, *** *p* < 0.001, and **** *p* < 0.0001.

LPS was dissolved in 0.9% normal saline and then injected intraperitoneally at a dose of 7 mg/kg for 3 days to establish the ALI model. VK2 was administered orally by gavage. The VK2 powder was placed in soybean oil (DMSO was not used throughout) and shaken on a vortex machine for 5 min to uniformly mix it into a liquid with a concentration of 1.5 mg/mL. The subsequent VK2 administration was divided into high and low doses, and the intragastric volumes were 200 μL and 20 μL, respectively. Before LPS treatment, the VK2 treatment groups had VK2 pre-administered by gavage for 7 days, while the control and model groups were given the same volume of solvent solution. In the positive control group, mice were administrated DEX (5 mg/kg, dissolved in saline) for 7 days after LPS injection. The mouse treatments are shown in Figure 1A. Twenty-four hours after the administration of saline or drug administration, mice were deeply anesthetized with tribromoethanol and then sacrificed, and the lungs and serum were collected for further study.

### 2.3. Histopathological Analysis of Lungs

Lung tissues were washed with pre-cooled phosphate buffered saline (PBS), fixed in 4% (*w*/*v*) paraformaldehyde, dehydrated in ethanol, embedded in paraffin, and sliced into 4 μm sections for hematoxylin–eosin (H&E) staining. As mentioned earlier, a semi quantitative scoring system is used to assess the severity of lung injury [31].

### 2.4. Detection of SOD and T-AOC Activities, and Ca^2+^ Level in Lung Tissue

Lung homogenate (10%, *w*/*v*) was obtained by homogenizing the lung tissue with normal saline. The supernatant was collected for the measurements of SOD, T-AOC, and the calcium (Ca^2+^) level using appropriate kits, following the manufacturer’s instructions.

### 2.5. Western Blot Analysis

Proteins from lung tissues were isolated using RIPA lysate buffer and the concentrations were determined by the BCA assay. Denatured proteins were separated by sodium dodecyl sulfate polyacrylamide gel electrophoresis (SDS-PAGE) and then transferred to polyvinulidene difluoride (PVDF) membranes. After blocking with 5% fat-free milk for 1 h at room temperature, the membranes were incubated with the primary antibodies at 4 °C overnight. The antibodies involve various cell signaling molecules such as pro-inflammatory factor (tumor necrosis factor alpha, TNF-α, WL01581, 1:2000), anti-inflammatory factor (interleukin-10, IL-10, LV-A12255, 1:1000), apoptotic marker (BCL2-associated X, Bax, ab243140, 1:500), anti-apoptotic marker (B-cell lymphoma-2, Bcl-2, ab32124, 1:1000), TJP markers (ZO-1, 21773-1-AP, 1:10,000 and occludin, WL01996, 1:1000), mitochondrial quality control markers (PINK1, 23274-1-AP, 1:600; Parkin, ET1702-60, 1:1000; Mfn2, 12186-1-AP, 1:5000 and PGC1α, ET1702-96, 1:1000), and autophagy and pathway markers (microtubule-associated proteins 1A and 1B, LC3, 18725-1-AP, 1:1000; Beclin1, WL02508, 1:1000; phosphatidylinositol 3-kinase, PI3K, WL03380, 1:1000 and protein kinase B, AKT, WL0003b, 1:1000). Next, the membranes were incubated with the secondary antibodies (Santa Cruz Biotechnology, Dallas, TX, USA) for 1 h at room temperature. Finally, the immunoblot was visualized using HRP-ECL reagent (Beyotime, Haimen, China) with the gel imaging system (Bio-Rad, Hercules, CA, USA).

### 2.6. Data Analysis

Data were in the form of the mean ± standard error of the mean (SEM). One-way analysis of variance (ANOVA) was used for statistical comparisons followed by the Kruskal–Wallis rank sum test for multiple groups with the assistance of GraphPad Prism Program (Version 7.04). Significance was set at * *p* < 0.05, ** *p* < 0.01, *** *p* < 0.001, and **** *p* < 0.0001.

## 3. Results

### 3.1. VK2 Mitigated LPS-Induced Lung Injury

The mouse treatments are shown in Figure 1A. The H&E staining of lung tissue showed that LPS induced significant lung injury, while the pretreatment with VK2 significantly mitigated LPS-induced lung injury (Figure 1B,C). As is shown in Figure 1D, 25% of the LPS-induced mice died after administration of DEX, but none of the mice died in the other groups. Dexamethasone-induced death in mice may be due to its inhibition of the early immune response in mice as an immunosuppressant. This is consistent with previous studies. In the early stages of the disease, a strong immune response is essential for survival. Some studies have shown that the use of dexamethasone in humanized mice with COVID-19 in the early stage of infection can lead to the death of mice [32]. Another study has shown that the use of dexamethasone in ALI mice also has the occurrence of death [33]. These results indicated that both DEX and VK2 alleviate LPS-induced lung injury, but VK2 was safer and had fewer side effects.

### 3.2. VK2 Alleviates LPS-Induced Lung Inflammation, Apoptosis, and TJ Injury

**Figure 2:**
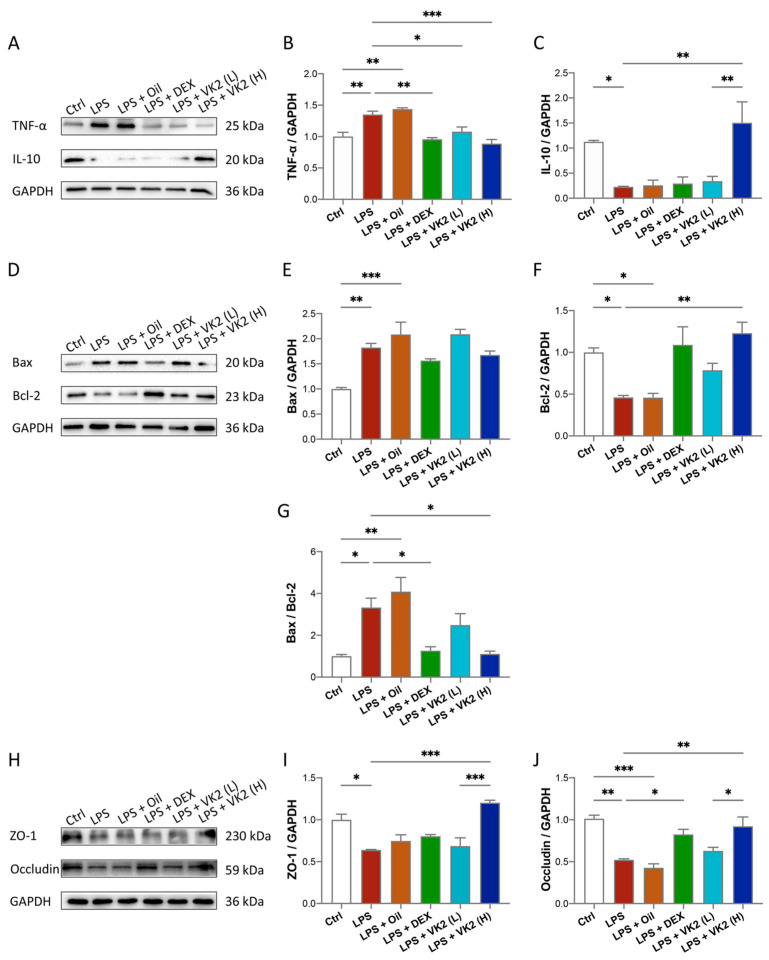
VK2 alleviates inflammation, apoptosis, and TJ injury in LPS-induced acute lung injury. (**A**) The protein expression levels of TNF-α and IL-10 were detected by immunoblotting. (**B**,**C**) Quantitative analysis of TNF-α and IL-10 was performed using Image J software (1.8.0) with glyceraldehyde-3-phosphate dehydrogenase (GAPDH) as an internal reference. (**D**) The protein expression levels of BAX and Bcl-2 were detected by immunoblotting. (**E**–**G**) Quantitative analysis of BAX, Bcl-2, and the ratio of BAX/Bcl-2 was performed using Image J software with glyceraldehyde-3-phosphate dehydrogenase (GAPDH) as an internal reference. (**H**) The protein expression levels of ZO-1 and occludin were detected by immunoblotting. (**I**,**J**) Quantitative analysis of ZO-1 and occludin was performed using Image J software with glyceraldehyde-3-phosphate dehydrogenase (GAPDH) as an internal reference. The value represents the mean ± standard error; * *p* < 0.05, ** *p* < 0.01, and *** *p* < 0.001.

In our study, we explored the effect of VK2 on inflammation, apoptosis, and TJ proteins. The results of TNF-α are shown in Figure 2A,B. Compared with the control group, the expression of TNF-α in the LPS group was significantly increased, by approximately 1.35-fold (*p* = 0.0077). Compared with the LPS group, the expression of TNF-α in the high-dose-VK2 intervention group was significantly decreased, which was approximately 0.65-fold greater than that of the LPS group (*p* = 0.0008). The results of IL-10 are shown in Figure 2A,C. Compared with the control group, the expression of IL-10 in the LPS group was significantly reduced by approximately four-fifths (*p* = 0.0476). Compared with the LPS group, the expression of IL-10 in the high-dose-VK2 group was significantly increased, which was approximately 6.8-fold greater than that of the LPS group (*p* = 0.0044). Apoptosis involves multiple signaling pathways, with the core changes being the activation of pro-apoptotic and downregulation of anti-apoptotic proteins. Compared with the control group, the level of the pro-apoptotic protein Bax (Figure 2D,E) in the LPS group increased by approximately 1.8-fold (*p* = 0.0039), and the level of the anti-apoptotic protein Bcl-2 (Figure 2D,F) decreased to approximately 0.5-fold (*p* = 0.0498). Compared with the LPS group, the Bcl-2 level was increased by approximately 2.6-fold (*p* = 0.0046) after high-dose-VK2 treatment. At the same time, compared with the control group, the ratio of Bax/Bcl-2 (Figure 2G) in LPS-treated mice increased by approximately 3.3-fold (*p* = 0.0176). Compared with LPS-treated mice, VK2 could downregulate the ratio of Bax/Bcl-2 to approximately one-third (*p* = 0.0018). Similarly, Western blot analysis results showed that LPS impaired the expression of ZO-1 (Figure 2H,I) and occludin (Figure 2H,J) proteins, which were reduced to approximately 0.6-fold (*p* = 0.0102) and 0.5-fold (*p* = 0.0011) compared with the control group, while high-dose VK2 could significantly restore the expression of ZO-1 and occludin proteins compared with the LPS group. They were increased to approximately 2-fold (*p* = 0.0002) and 1.7-fold (*p* = 0.0059), respectively. Collectively, these findings demonstrate that VK2 protects the integrity of TJs and exhibits anti-inflammatory and anti-apoptotic effects in ALI.

### 3.3. VK2 Inhibits Oxidative Stress and Maintains Calcium Homeostasis by Regulating Mitochondrial Function in LPS-Induced ALI

**Figure 3:**
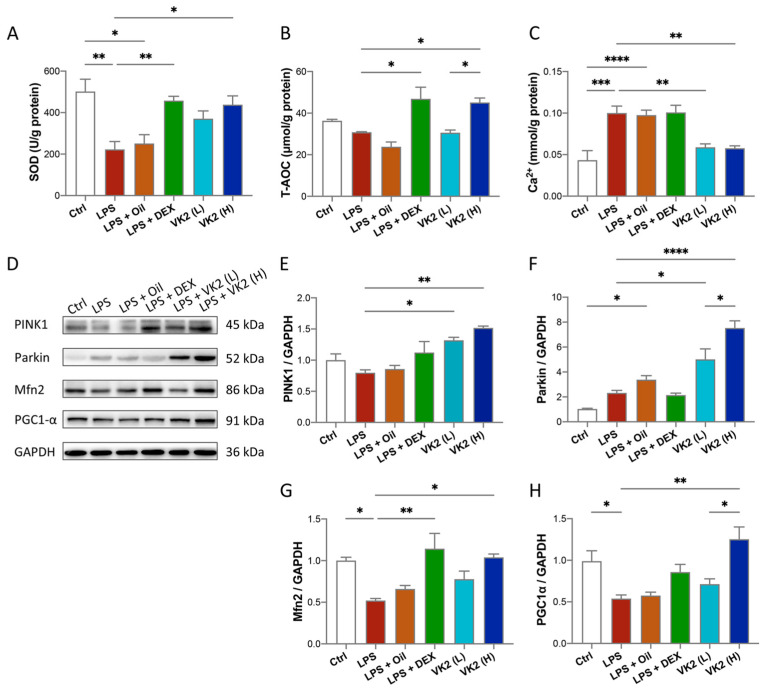
VK2 inhibits oxidative stress and maintains calcium homeostasis by regulating mitochondrial function in LPS-induced ALI. (**A**) SOD, (**B**) T-AOC, and (**C**) Ca^2+^ levels were measured with kits in lung tissue. (**D**) The protein expression levels of PINK1, Parkin, Mfn2, and PGC1-α were detected by immunoblotting. (**E**–**H**) Quantitative analyses of PINK1, Parkin, Mfn2, and PGC1-α were performed using Image J software with glyceraldehyde-3-phosphate dehydrogenase (GAPDH) as an internal reference. The value represents the mean ± standard error; * *p* < 0.05, ** *p* < 0.01, *** *p* < 0.001, and **** *p* < 0.0001.

LPS challenge increases pulmonary oxidative stress that is confirmed by lower levels of some antioxidants [34]. Therefore, we investigated the effect of VK2 on the lung levels of some oxidative-stress-related indicators in the ALI model. Our results showed significantly lower levels of SOD activity (*p* = 0.0024, Figure 3A) in the LPS group when compared with comparable findings of the control group. On the contrary, VK2 pretreatment in the LPS group at 15 mg/kg, but not at 1.5 mg/kg, significantly and suitably improved level of SOD (*p* = 0.0209, Figure 3A) and T-AOC (*p* = 0.0246, Figure 3B). Calcium is an important second messenger and it is widely recognized that intracellular calcium (Ca^2+^) oscillations are vital in ALI, leading to reduced integrity of the lung epithelial and endothelial barriers and increased lung inflammation [35]. As is presented in Figure 3C, LPS evoked a significant Ca^2+^ increase compared with the control group (*p* = 0.0003), while VK2 pretreatment significantly decreased Ca^2+^ levels (*p* = 0.0035). Mitochondria are involved in immunological responses, redox signaling, apoptosis, autophagy, and maintaining calcium homeostasis [36]. Western blot analysis results showed that compared with the control group, the expression levels of Mfn2 (Figure 3D,G) and PGC1-α (Figure 3D,H) in the LPS group were significantly decreased by approximately 0.5-fold (*p* = 0.0264 and 0.0492). Compared with LPS group, VK2 administration significantly increased the expression of PINK1 (Figure 3D,E), Parkin (Figure 3D,F), Mfn2 (Figure 3D,G), and PGC1-α (Figure 3D,H) to approximately 1.9-fold (*p* = 0.0013), 3.2-fold (*p* < 0.0001), 2-fold (*p* = 0.0158), and 2.5-fold (*p* = 0.0019), respectively. These results indicated that VK2 could inhibit oxidative stress and calcium overload by improving mitochondrial impairment.

### 3.4. VK2 Alleviates LPS-Induced Lung Autophagy

**Figure 4:**
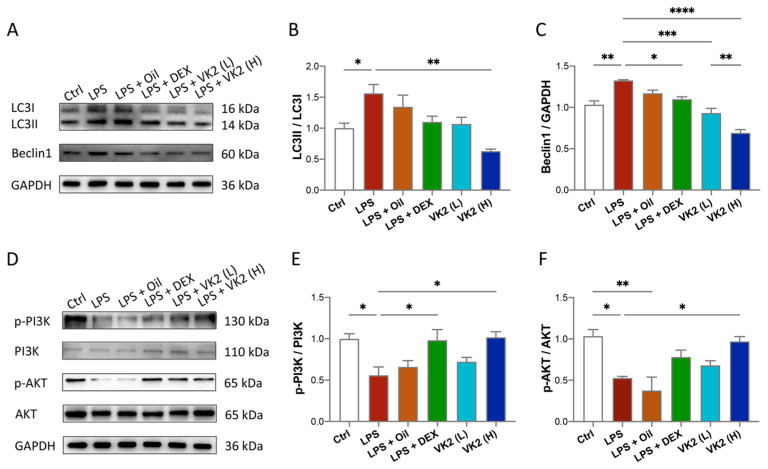
VK2 alleviates LPS-induced lung autophagy through PI3K/AKT pathway. (**A**) The protein expression levels of LC3 and Beclin1 were detected by immunoblotting. (**B**,**C**) Quantitative analysis of the ratio of LC3II/LC3I and Beclin1 were performed using Image J software with glyceraldehyde-3-phosphate dehydrogenase (GAPDH) as an internal reference. (**D**) The protein expression levels of p-PI3K, PI3K, p-AKT, and AKT were detected by immunoblotting. (**E**,**F**) Quantitative analysis of the ratio of p-PI3K/PI3K and p-AKT/AKT were performed using Image J software with glyceraldehyde-3-phosphate dehydrogenase (GAPDH) as an internal reference. The value represents the mean ± standard error; * *p* < 0.05, ** *p* < 0.01, *** *p* < 0.001 and **** *p* < 0.0001.

To investigate whether VK2 could alleviate LPS-induced lung autophagy, the anti-autophagic effect of VK2 on ALI mice was evaluated. Our data showed that compared with the control group, the expression of LC3II (Figure 4A,B) and Beclin1 (Figure 4A,C) in the LPS group was significantly increased to approximately 1.5-fold (*p* = 0.0492) and 1.3-fold (*p* = 0.0019), respectively. However, compared with the LPS group, VK2 significantly downregulated the expression of LC3II (Figure 4A,B) and Beclin1 (Figure 4A,C) to approximately 0.4-fold (*p* = 0.0013) and 0.52-fold (*p* < 0.0001), respectively. In view of the contributions of the PI3K/AKT pathway to autophagy, we further measured PI3K, p-PI3K, AKT, and p-AKT levels in the lung tissue. Our results showed that compared with the control group, LPS significantly reduced the ratio of p-PI3K/PI3K (Figure 4D,E) and p-AKT/AKT (Figure 4D,F) by approximately 0.5-fold (*p* = 0.0312 and 0.0159). Compared with the LPS group, VK2 significantly increased the ratio of p-PI3K/PI3K (Figure 4D,E) and p-AKT/AKT (Figure 4D,F) to approximately 1.8-fold (*p* = 0.0242 and 0.0379), respectively, suggesting that VK2 may attenuate LPS-induced autophagy through activating the PI3K/AKT pathway.

## 4. Discussion

VK2 is a natural product from a large number of bacteria, and it is lipophilic and can easily transfer to the host intestinal cells [37]. It has shown protective effects against inflammation, oxidant stress, apoptosis, autophagy, and mitochondrial damage. Studies on VK2 in ALI are few. Our study found that VK2 regulated inflammation, apoptosis, and TJ protein damage in LPS-induced ALI through the inhibition of the mitochondrial damage and autophagy.

Dexamethasone is a non-specific immunosuppressant that has been widely used to treat various diseases, including autoimmune diseases, allergies, eye diseases, cancer, pneumonia, and most recently, COVID-19 [38]. However, the use of dexamethasone is often limited in clinical practice due to its poor water solubility. When administered by the systemic route, it can cause serious side effects, such as hypertension, peptic ulceration, hyperglycemia, and water electrolysis disorder [39,40]. Although it has been used in the treatment of COVID-19, studies have shown that patients treated with dexamethasone have lower survival rates and longer recovery times than with high-dose methylprednisolone [41]. In another randomized clinical trial of patient in intensive care unit with COVID-19-related acute hypoxic respiratory failure, high-dose dexamethasone did not significantly improve the 60-day survival rate [42]. Therefore, we speculate that although dexamethasone has a certain therapeutic effect on pneumonia, it also has obvious side effects. An acute oral toxicity test in mice and a subchronic toxicity study in rats showed the safety of VK2 for human consumption at typical supplemental doses [43]. In addition, a US Pharmacopeial Convention safety evaluation of MK-7 indicated that MK-7, when consumed as a dietary supplement, did not pose any serious health risk or other public health concerns [25]. In this study, we found that DEX administration could cause the death of mice, but VK2 could not, suggesting that VK2 was safer and had fewer side effects compared to DEX (Figure 1D).

Inflammation, apoptosis, and the disruption of intercellular TJPs are the main reasons for ALI [44]. Maintaining the good condition of TJs and inhibiting inflammation and apoptosis are all important for the prognosis of ALI. VK2 (MK-7) has been shown to have anti-inflammatory and anti-apoptosis effects in Alzheimer’s disease [45,46,47]. It can also promote the expression of TJPs to protect the intestinal barrier function in colitis [48]. However, its role in LPS-induced ALI has not been clarified. According to our study, VK2 pretreatment obviously downregulated pro-inflammatory and pro-apoptotic protein expression and upregulated anti-inflammatory and anti-apoptotic protein expression in LPS-induced ALI. And, we found that VK2 markedly restored TJP expression to maintain the integrity of the lung epithelial and endothelial barriers in LPS-induced ALI (Figure 2).

It has been confirmed that oxidative stress contributes to the pathogenesis of ALI, such as via inflammation, apoptosis, and endothelial barrier dysfunction [49,50]. Thus, we tested the SOD and T-AOC levels in the lung tissue in each group, and the results confirmed that VK2 might have a protective effect against oxidative stress in ALI. Several lines of evidence suggest that oxidative stress occurs as a consequence of the mitochondrial dysfunction [51]. Therefore, we speculate that the protection against oxidative stress by VK2 is related to the improvement in mitochondrial function. In order to maintain mitochondrial homeostasis and ensure mitochondrial function, multiple pathways such as mitochondrial biosynthesis, mitochondrial fusion, and mitophagy are involved in mitochondrial quality control [52]. Our results showed that treatment with VK2 improved the mitochondrial function, and increased the PINK1/Parkin signaling pathway of mitophagy, the fusion protein Mfn2, and mitochondrial biogenesis regulator PGC-1α expression. Previous studies have shown that VK2 could reinstate mitophagic activities by upregulating PINK1 and Parkin proteins [30,53]. Furthermore, as a mitochondrial electron carrier, VK2 could rescue PINK1-deficiency-induced mitochondrial dysfunction [54]. Additionally, VK2 promoted mitochondrial fusion and biogenesis by increasing the expression of Mfn2 and PGC-1α [30]. Therefore, our results suggest that mitochondrial quality control may be an important mechanism for VK2 to improve mitochondrial function in ALI.

The calcium in mitochondria is a double-edged sword. Although low levels of calcium are crucial for maintaining most excellent ATP production speed rates, overcoming the extreme levels of mitochondrial calcium retention can lead to mitochondrial dysfunction [55]. So far, few studies have reported on the effects of VK2 on calcium homeostasis regulation. According to our results, the calcium level was significantly decreased under VK2 pretreatment compared with that in the LPS group, suggesting that pretreatment with VK2 inhibited calcium overload in ALI.

Autophagy is highly conserved self-digestion process of cells that leads to the degradation of macromolecular substances and organelles that are destroyed, denatured, senescent, and under the influence of external environmental factors by lysosomes [56]. The pathogenesis of ALI is complex, and important cellular and molecular events include abnormal autophagy [57]. Excess autophagy results in autophagic cell death [58]. Although more and more studies have shown that VK2 induces autophagy in cancer [59,60,61], a previous study has indicated that VK3 reduced pancreatic inflammation in acute pancreatitis by inhibiting the impaired autophagy [29]. Our study found that excessive autophagy was activated in LPS-induced ALI. We further found that the pretreatment of VK2 suppressed excessive autophagy. Furthermore, in terms of molecular mechanisms, VK2 activated the PI3K/AKT pathway, which is one of the most important pathways that controls autophagy [62].

In a previous study [63], we also carried out some protective studies of vitamin K2 on acute lung injury. The conclusions of the two articles are consistent, both of which reflected the fact that vitamin K2 can protect ALI mice by interfering with inflammation and apoptosis. The previous article introduced that VK2 acts on three aspects of the TLR4-MAPK signaling pathway, ferroptosis-related factors GPX4 and HO-1, and the vitamin-K-dependent protein (VKDP) MGP. This article describes the role of VK2 in terms of two aspects: the PI3K/AKT signaling pathway and calcium homeostasis. The existing literature shows that vitamin K2 can act on a variety of VKDPs to play different functions [37]. For example, VKDP GAS6 can bind to the PI3K/AKT signaling pathway [64]. Among them, some VKDPs are transmembrane proteins, such as TGM3 and PRGP1, which may be involved in the signaling pathway [37]. TLR4, as a VKDP membrane protein, is likely to have a combined effect. In addition, vitamin K can also form a non-classical vitamin K cycle with its reductase enzyme FPS1 to effectively inhibit ferroptosis [65]. VK2 binds to VKDP to exert effects on four aspects of the ALI-related TLR4–MAPK signaling pathway, DES, the PI3K/AKT signaling pathway, and calcium homeostasis (VKDP may bind to calcium channel proteins). VK2 inhibits ferroptosis through a non-classical vitamin K cycle pathway.

## 5. Conclusions

**Figure 5:**
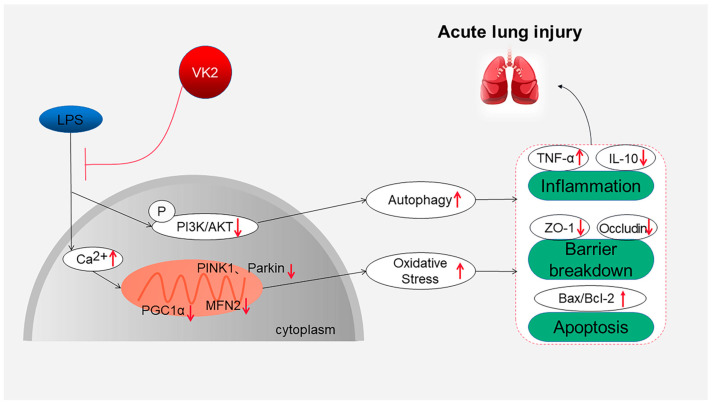
Overview of the effects and mechanisms of VK2 in LPS-induced ALI. On the one hand, VK2 leads to ensuring mitochondrial function, thereby inhibiting oxidative stress, and further inhibiting inflammation, apoptosis, and tight junction protein loss. On the other hand, VK2 inhibits excessive autophagy by upregulating the PI3K/AKT pathway, thereby reducing inflammation, apoptosis, and tight junction protein loss.

Overall, this study suggests that VK2 plays an important protective role in regulating inflammation, cell apoptosis, and TJ injury in LPS-induced ALI by inhibiting mitochondrial dysfunction and excessive autophagy, as shown in Figure 5. These findings provide a theoretical basis for the clinical application of VK2 in ALI therapy and the development of treatment strategies involving VK2. The limitation of this study is that further research is needed to investigate the mechanism and clinical applications of VK2. We will continue to carry out related studies in depth in the future.

## Data Availability

Data are available on reasonable request.
